# Controlled Localization of Functionally Active Proteins to Inclusion Bodies Using Leucine Zippers

**DOI:** 10.1371/journal.pone.0097093

**Published:** 2014-06-04

**Authors:** Su-Lim Choi, Sang Jun Lee, Soo-Jin Yeom, Hyun Ju Kim, Young Ha Rhee, Heung-Chae Jung, Seung-Goo Lee

**Affiliations:** 1 Biochemicals and Synthetic Biology Research Center, KRIBB, Yuseong-gu, Daejeon, Korea; 2 Department of Bioscience and Biotechnology, Chungnam National University, Yuseong-gu, Daejeon, Korea; 3 Infection and Immunity Biology Research Center, KRIBB, Yuseong-gu, Daejeon, Korea; 4 Korea University of Science and Technology, Yuseong-gu, Daejeon, Korea; University of Crete, Greece

## Abstract

Inclusion bodies (IBs) are typically non-functional particles of aggregated proteins. However, some proteins in fusion with amyloid-like peptides, viral coat proteins, and cellulose binding domains (CBDs) generate IB particles retaining the original functions in cells. Here, we attempted to generate CBD IBs displaying functional leucine zipper proteins (LZs) as bait for localizing cytosolic proteins in *E. coli*. When a red fluorescent protein was tested as a target protein, microscopic observations showed that the IBs red-fluoresced strongly. When different LZ pairs with K_D_s of 8–1,000 µM were tested as the bait and prey, the localization of the red fluorescence appeared to change following the affinities between the LZs, as observed by fluorescence imaging and flow cytometry. This result proposed that LZ-tagged CBD IBs can be applied as an *in vivo* matrix to entrap cytosolic proteins in *E. coli* while maintaining their original activities. In addition, easy detection of localization to IBs provides a unique platform for the engineering and analyses of protein-protein interactions in *E. coli*.

## Introduction

Inclusion bodies (IBs) are dense, electron-refractile particles of aggregated proteins found in the cytoplasmic space of bacterial cells [1]. Hydrophobic heterologous proteins expressed at high levels in bacterial cells are likely to accumulate in IBs [2], [3]. IBs vary in diameter from 0.5–1.3 µm and are more dense (∼1.3 mg/mL) than many other cellular components, which make the particles easy to separate from disrupted cells by high-speed centrifugation for protein refolding [4], [5].

In general, the proteins in IBs are functionally inactive. However, recent studies have shown that they are not necessarily inactive, and some exhibit substantial levels of activity in *E. coli* [2], [3], [6]–[8]. For example, certain enzymes fused to a viral capsid protein or an ionic self-assembling peptide generated active IBs that had high levels of catalytic activity [6]–[8]. Accordingly, we found that a family II cellulose binding domain (CBD) from *Cellulomonas fimi* induced the formation of active IBs when fused with β-glycoside hydrolyzing enzymes. The enzymatic activity of these IBs was 30%–40% of that of the soluble enzymes [9]. In addition, a family IIIa CBD has also been used to form active IBs with high D-amino acid oxidase activity [10]. The family II CBD in IBs also exhibited significant binding affinity towards insoluble celluloses [9].

**Figure 1:**
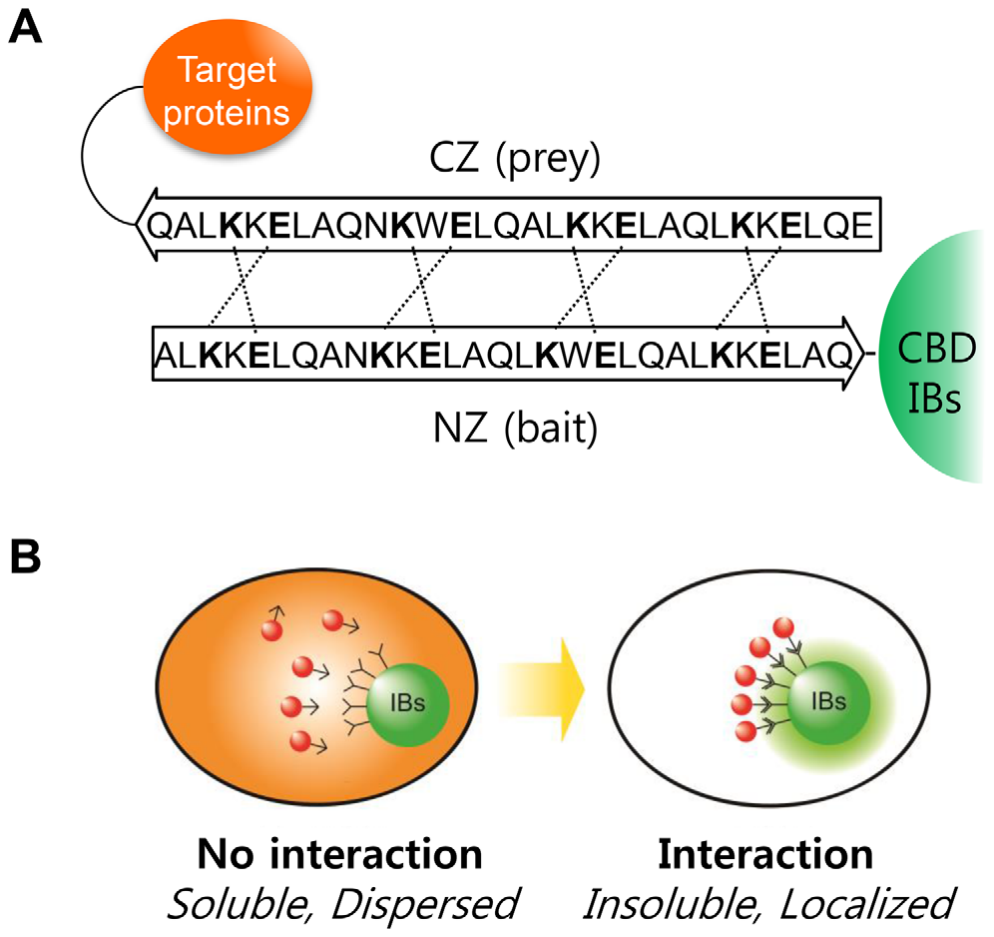
Controlled localization of functionally active proteins to inclusion bodies (IBs) using leucine zippers (LZs). **A.** Interactions between anti-parallel leucine zippers (LZ). Dashed lines indicate the charge-charge interactions of glutamic acid (E) and lysine (K). **B.** Representation of controlled localization in bacterial cell. The red fluorescent protein, which is dispersed throughout the cytosol, is localized to IBs by a specific molecular interaction.

In this study, the family II CBD from *C. fimi* was used to generate IBs displaying functional leucine zipper proteins (LZs) as bait for localizing soluble cytosolic proteins in *E. coli* (Fig. 1A). LZs are universal, two-stranded, α-helical heterodimers that are found in diverse DNA binding proteins and dimerization domains [11], [12]. Therefore, the heterodimer formation between LZs was expected to recruit soluble, functionally active proteins to IBs (Fig. 1B). As a soluble model protein, monomeric red fluorescent protein 1 (mRFP1) [13] was used to allow for rapid and quantitative analysis in living cells. Imaging and flow cytometric analyses showed that protein localization increased according to the binding affinity between the LZ proteins, consistent with the observations of a report that showed that dimerization of coil proteins caused the co-purification of soluble enzymes in IB fractions [14]. Our affinity-based localization of cytosolic proteins to active IBs is expected to be useful for many biotechnology applications: for example, the *in vivo* matrix can be used to localize enzymes for sequential reactions to the same locations in cells, thereby adjusting the local concentration of the enzymes and reducing intermediate loss through diffusion and side reactions [15]–[17]. In addition, as the localization of interacting proteins to IBs can be easily identified, this study provided a new platform for investigating protein-protein interactions in living cells, using fluorescence microscopy or flow cytometry [18].

## Materials and Methods

### Materials

The family II CBD was cloned from the exoglucanase (cex) of *Cellulomonas fimi* KCTC 9143. The *EGFP* gene was obtained from the commercial plasmid pEGFP-N1 (Clontech, Mountain View, CA, USA). The pRFP plasmid, which contains the gene for monomeric red fluorescent protein 1 (*mrfp*) [13], was a kind gift from Dr. R. Tsien (UCSD, USA). Genes encoding two anti-parallel LZs, used for the bait and prey, were cloned from pET11a-Z-NGFP and pMRBAD-Z-CGFP [12], respectively, which were provided by Dr. L. Regan (Yale University, USA). *E. coli* DH5α (Takara Bio, Ohtsu, Japan) and BL21(DE3) (Novagen, Gibbstown, NJ, USA) cells were used as the cloning host and the expression host, respectively. All restriction enzymes were purchased from Roche Applied Science (Indianapolis, IN, USA), and T4 DNA Ligase was purchased from Fermentas (Glen Burnie, MD, USA).

### DNA manipulation

All primers were synthesized by Bioneer Co. (Daejeon, Korea) (Table S1). The *EGFP* gene was amplified from pEGFP-N1, and then cloned into the *Nde*I and *Xho*I sites of pET21a (Invitrogen, Carlsbad, CA, USA) to yield pEGFP. The *EGFP-CBD* gene was prepared using overlap PCR and was inserted into the *Nde*I and *Hin*dIII sites of pET21a to yield plasmid pEGFP-CBD. The bait and prey LZs were fused to the *EGFP-CBD* and *mRFP* genes, respectively, by overlap extension PCR (Fig. S1). The resulting *bait-EGFP-CBD* and *prey-mRFP* genes were then inserted into pET21a to yield pCN20-CBD. pC20-CBD, a bait-less variant of pCN20-CBD was constructed by using *EGFP-CBD* instead of *bait-EGFP-CBD*. Four variants of the pCN20-CBD plasmid (pCN8-CBD, pCN31-CBD, pCN50-CBD, and pCN1000-CBD) were constructed by introducing known mutations into the prey moiety as shown in Table 1 [12], using a QuikChange mutagenesis kit (Stratagene, La Jolla, CA, USA).

**Table 1:** Amino acid sequences and affinity information of mutant CZs.

No.	CZ peptide	Mutations	K_D_ (µM)
CN8-CBD	EQLKKKLQALEKKLAQLEWKNQALEKELAQ	4/27	8
CN20-CBD	EQLEKKLQALEKKLAQLEWKNQALEKKLAQ	None	20
CN31-CBD	EQLEKKLQALEKKLAQLEWKNQALKKKLAQ	25	31
CN50-CBD	EQLEKKLQALKKKLAQLEWKNQALEKKLAQ	11	50
CN1000-CBD	EQLEKKLQALEKELAQLEWKNQALKKELAQ	13/25/27	1000

Mutation sites are underlined. The CZ peptide sequences and KD's were adopted from the results of Magliery TJ et al. [12].

### Protein expression and western blotting analysis

*E. coli* BL21(DE3) cells were cultivated at 37°C in LB medium containing ampicillin (50 µg/mL). Protein expression was induced with 0.5 mM IPTG when the cultures reached an OD_600_ of 0.5, and the cells were incubated for an additional 6 h. The cells were harvested by centrifugation at 16,300×*g* for 10 min and then disrupted by sonication on ice.

The protein expression was analyzed by SDS-PAGE and western blotting. Aliquots of cell lysates were electrophoresed on 12% SDS-polyacrylamide gels and electro-transferred to polyvinylidene fluoride membranes (Millipore, Billerica, MA, USA). The membranes were hybridized with an anti-GFP mouse antibody (Sigma-Aldrich, St. Louis, MO, USA) and an anti-groEL antibody as the internal standard (Abcam, Cambridge, MA, USA), followed by an HRP-conjugated anti-mouse IgG goat antibody (Bio-Rad, Hercules, CA, USA) prepared in TBST buffer (20 mM Tris-HCl, 100 mM NaCl, and 0.1% Tween-20, pH 7.5) containing 5% skimmed milk. The hybridized bands were identified by colorimetric detection using an Opti-4CN substrate kit (Bio-Rad).

### Imaging and fluorescence analyses

Cells were observed with an Axio Observer microscope (Carl Zeiss, Oberkochen, Germany) at ×1,000 magnification under differential interference contrast (DIC) imaging conditions. Fluorescence imaging was also performed using the same microscope fitted with a GFP filter (excitation BP 470/20, beam splitter FT 493, emission BP 505–530) and a rhodamine filter (excitation BP 546/12, beam splitter FT 580, emission LP 590) for EGFP and mRFP, respectively. Image acquisition and region-of-interest analyses were performed using MetaMorph software (Molecular Devices, Sunnyvale, CA, USA). At least 5 cells per image were selected and subjected to region-of-interest analyses. All ROI data were presented as means ± standard error of the mean.

### Flow cytometry

Flow cytometric analyses were performed using a FACSCalibur flow cytometer (BD Biosciences, Franklin Lakes, NJ, USA). The gate was set based on side scatter channel (SSC) and forward scatter channel (FSC) parameters, and the EGFP and mRFP signals were detected using FL1 (530/30 nm) and FL2 (585/42 nm) photomultiplier tubes (PMTs), respectively. The overlap of the EGFP and mRFP signals was minimized using a compensation option. A total of 10^4^ cells were counted for each sample and the data were collected using BD CellQuest Pro software (version 4.0.2; BD Biosciences). Cell sorting was performed using a FACSAria Cell Sorter (BD Biosciences) at KRIBB, Jeonbuk Branch (Jeongeup, Korea).

### Electron microscopy and Zeta-potential analysis

For SEM imaging, purified CBD-IBs were fixed in a mixture of 2.5% paraformaldehyde and 2.5% glutaraldehyde in a 100-mM sodium phosphate buffer (pH 7.2) for 2 h, post-fixed with 1% osmium tetroxide in the same buffer for 1 h, dehydrated in graded ethanol, substituted with isoamyl acetate, and then critical point dried in CO_2_. The samples were then coated with gold in a SC502 sputter coater (Quorum Technologies Ltd, East Sussex, UK) and observed under a Quanta 250 FEG scanning electron microscope (FEI, Hillsboro, OR, USA) at KRIBB (Daejeon, Korea).

The size and zeta-potential of the EGFP-IBs were measured using a Malvern Zetasizer Nano ZS (Malvern Instruments, Malvern, UK) at the National Nanofab Center (Daejeon, Korea). The protein solution was diluted with 10 mM Tris-HCl (pH 8.0), and 0.75 mL of the diluted solution was added to disposable zetasizer cuvettes for the measurements. The experiments were performed in triplicate and the data were processed using Zetasizer Nano software (version 6.01; Malvern Instruments).

## Results

### Generation of functional IBs

**Figure 2:**
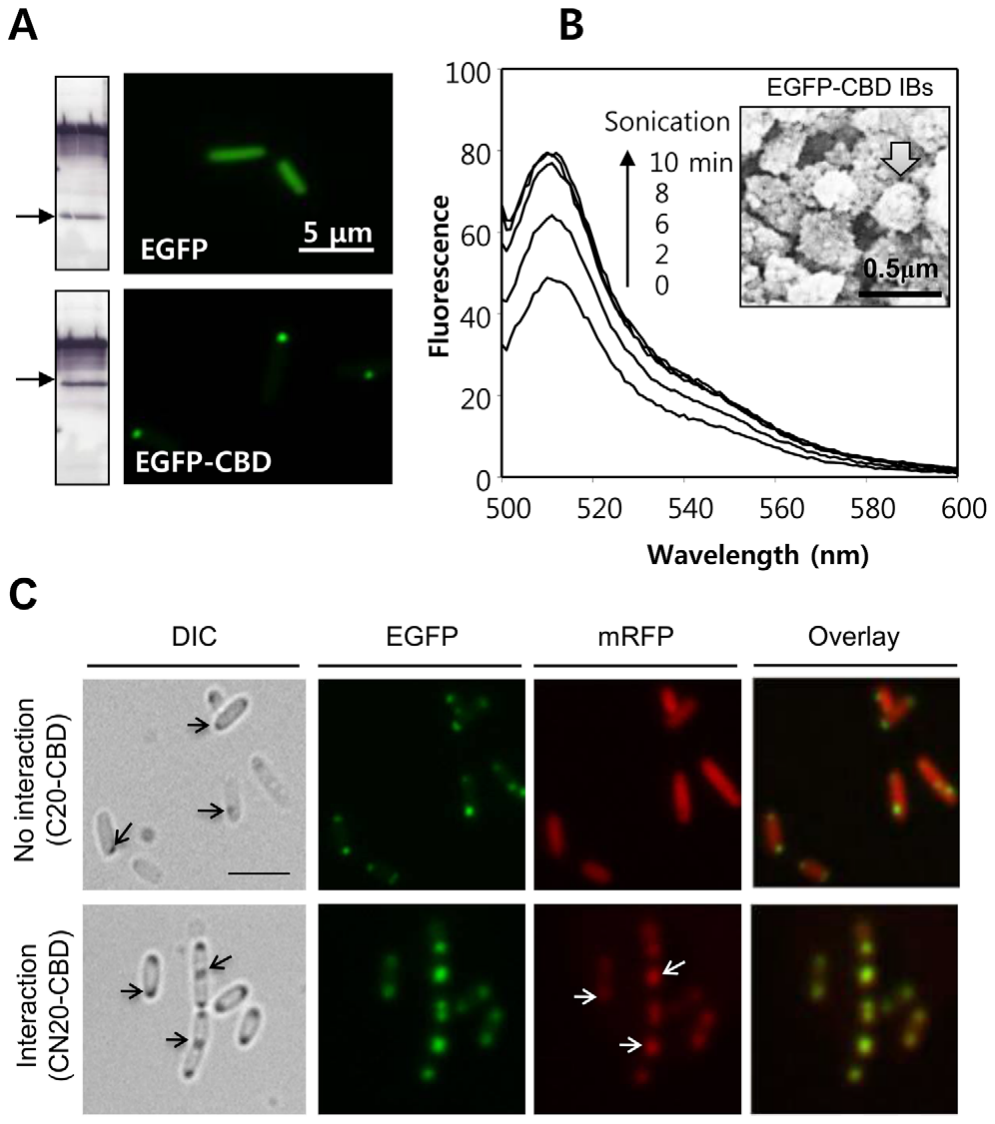
Microscopic observation of controlled localization to CBD IBs. **A.** Imaging expressed EGFP and EGFP-CBD in *E. coli*. The left panel represents the western blot images after treatment with anti-GFP and anti-GroEL antibodies. The EGFP band is indicated by the black arrows. Scale bar = 5 µm. **B.** Increased fluorescence following sonication of EGFP-CBD IBs. *E. coli* cells expressing EGFP-CBD were treated by sonication in a Tris buffer (50 mM Tris-HCl, pH 8.0, 200 mM NaCl) and the fluorescence intensity analyzed using a Cary Eclipse fluorometer. The inset represents a SEM image of the EGFP-CBD IBs. **C.** Microscopic images of *E. coli* cells with no interaction (top) and interaction (bottom) between LZs. Scale bar = 5 µm.

The CBDs include three to four aromatic residues that are exposed to bulk liquid on the surface of the protein (http://www.pdb.org; PDB ID: 1exg) [19], which may cause rapid aggregation of the protein. As previously mentioned, C-terminal fusions of the family II CBD from *C. fimi* formed active IBs retaining 30%–40% of the original activity while maintaining the ability to bind insoluble celluloses [9]. In the current study, *E. coli* cells expressing a fusion of the CBD with EGFP exhibited one or two fluorescent IBs in microscopic images (Fig. 2A). When cells expressing either EGFP or EGFP-CBD were compared by flow cytometry, the fluorescence intensity of the EGFP-CBD cells was estimated to be 10%–20% of that in cells expressing soluble EGFP (Fig. S2), although the expression of both proteins (as detected by western blotting) was similar. When the EGFP-CBD cells were sonicated in Tris buffer (50 mM Tris-HCl, pH 8.0 and 200 mM NaCl) to break the IBs into smaller pieces, the fluorescence intensity increased up to 2 folds in proportion to the sonication time (Fig. 2B). Therefore, the IBs are estimated to contain higher amounts of properly folded/native-like protein than that observed in flow cytometry. The low detection of fluorescence in IBs is discussed further in the Discussion section.

### Localization of soluble proteins to IBs

The possibility of active IBs as a matrix to recruit soluble cytosolic proteins was tested by displaying a bait LZ that can bind to prey LZs in cytosol (Figs. 1A and 1B). LZ is a super-secondary structure that generates adhesion forces between α-helices. A single LZ consists of multiple leucine residues at approximately 7-residue intervals, which forms an amphipathic alpha helix with a hydrophobic region on one side. This hydrophobic region provides an area for dimerization, allowing the motifs to combine. Therefore, fusion proteins tagged with prey LZs may form a two-stranded α-helical coiled-coil heterodimer with the bait LZ in active IBs (Fig. 1A). A monomeric red fluorescent protein 1 (mRFP1) was used as a model prey protein to take advantage of its easy detection in living cells. The *bait-EGFP-CBD* and *prey-mRFP1* genes were cloned into pET21a in a polycistronic manner to balance the relative expression of the bait and prey. When these bait and prey proteins were co-expressed in *E. coli* cells, the red fluorescence was clearly localized to the IBs (lower row in Fig. 2C), whereas the red fluorescence remained dispersed in cells without the bait LZ (upper row in Fig. 2C), showing that localization was dependent on the bait LZ.

**Figure 3:**
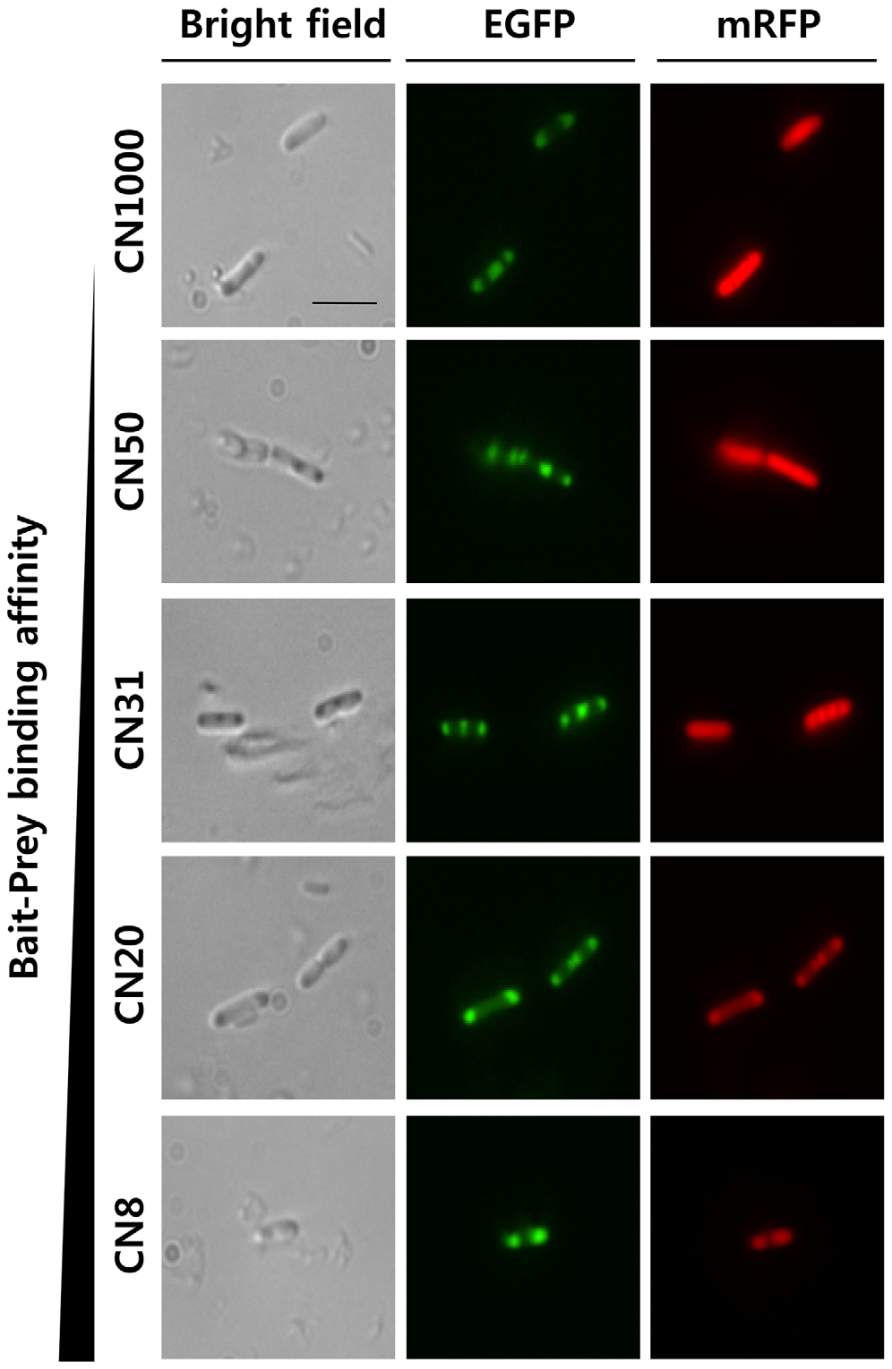
Effects of binding affinity between LZs. Microscopic images of *E. coli* cells containing LZ pairs with varying affinities (K_D_ = 8, 20, 31, 50, and 1,000 µM). Scale bar = 5 µm.

**Figure 4:**
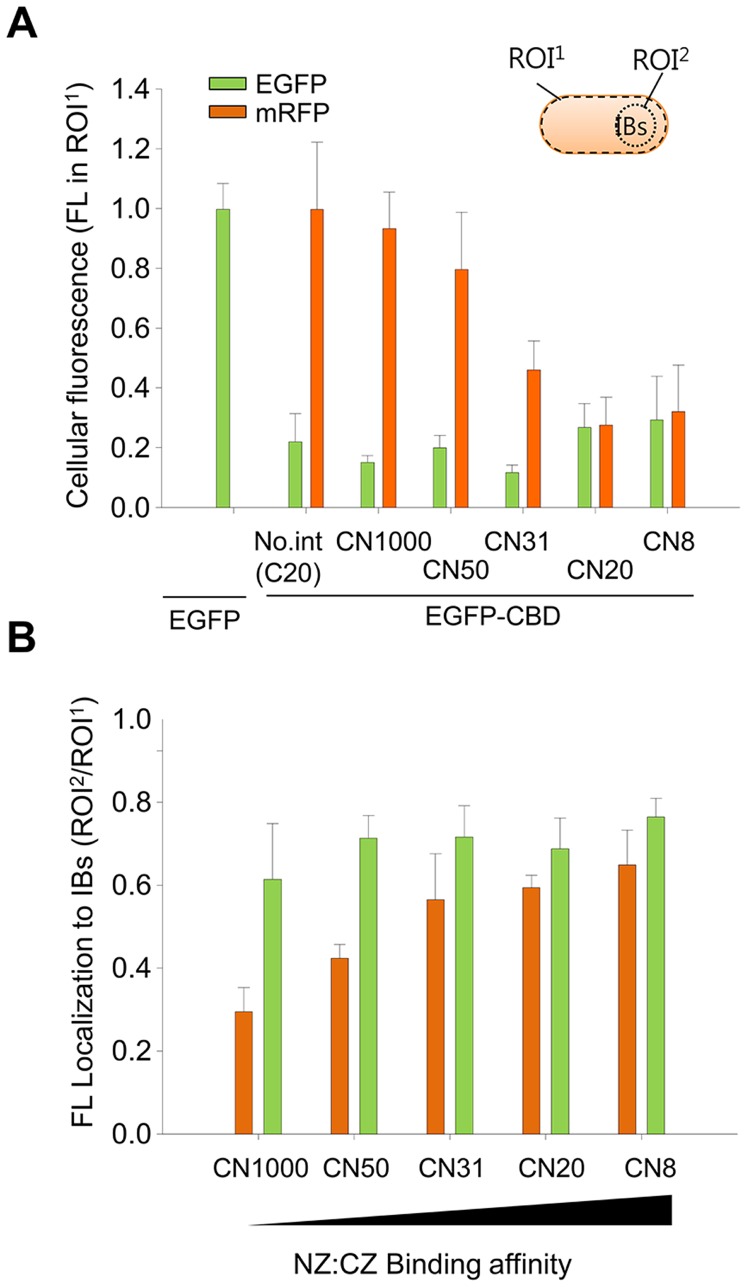
Region of interest (ROI) analysis of microscopic images. **A.** Cellular fluorescence decreased in proportion to the binding affinity between LZs in IBs. **B**. Comparison of localization yield to IBs. The fluorescence in IBs was normalized to the total cellular fluorescence, ROI^2^/ROI^1^, where ROI^1^ is the cellular area and ROI^2^ is the IB area of the cell. More than five cells per image were examined for the ROI analysis. Error bars show the standard deviations from 5 independent measurements of the cells.

Next, the effect of LZ binding affinity was investigated using different combinations of LZs (shown in Table 1). The leucine residue is essential for duplex formation, whereas ionic interactions between oppositely charged residues affected binding affinity. We examined five different bait and prey pairs that were designed by Magliery et al. [12] with K_D_ values of 8, 20, 31, 50, and 1,000 µM. As anticipated, more red fluorescence was observed to localize to the IBs when bait-prey pairs with smaller K_D_ values were used for the co-expression experiments (Fig. 3). When region-of-interest (ROI) analysis was applied to the cellular images (Fig. 4A), red fluorescence in cytosol decreased as the prey-mRFP1 protein localized to the IBs. Consequently, the mean yield of localization to IBs, ROI^2^ *vs*. ROI^1^, was calculated from at least five single cell images and a high yield of 0.65 was estimated for CN8 (K_D_ = 8 µM), which was nearly the same as the mean yield for EGFP-CBD (Fig. 4B). The yield for CN1000 (K_D_ = 1,000 µM) was approximately 0.30. Therefore, the higher the affinity of the bait for the prey, the more prey-mRFP localized to the IBs. In all the experiments, the expression levels of the bait-EGFP-CBD and prey-mRFPs were similar (as shown by SDS-PAGE analyses) (Fig. S3).

**Figure 5:**
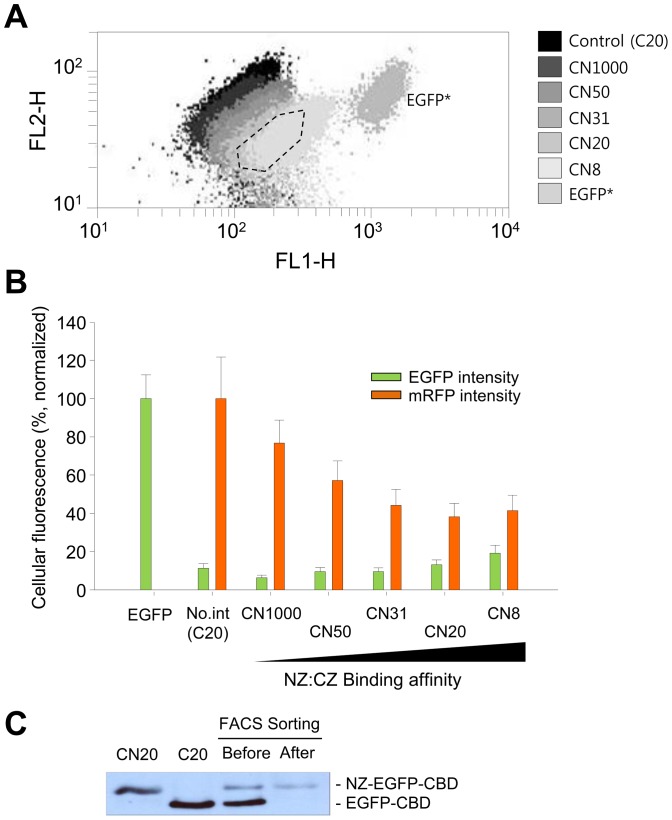
Flow cytometric analyses of controlled localization to IBs. **A.** FL1 *vs.* FL2 dot plot of cells containing LZ pairs with varying binding affinities (K_D_ = 8, 20, 31, 50, and 1,000 µM). EGFP* indicates the *E. coli* cells that expressed soluble EGFP and soluble mRFP (the controls). The dashed line indicates the sorting gate. **B.** Comparison of the fluorescence intensity of cells expressing different leucine zipper pairs. **C**. Western blot analysis of CN20-CBD cells sorted by the FACSAria. Lane 1, CN20-CBD cells; lane 2, C20-CBD cells; lane 3, a mixture of CN20-CBD and C20-CBD cells before sorting; lane 4, a mixture of CN20-CBD and C20-CBD cells after sorting. Error bars show the standard deviations from 5 independent measurements of the cells.

The localization of red fluorescence to IBs was also investigated by flow cytometry. When the cytometric results were drawn on FL1 *vs.* FL2 dot plots, the mRFP intensity (FL2) decreased as the binding affinity increased (Fig. 5A), whereas the EGFP intensity (FL1) increased. For example, the mean intensity of mRFP for the CN8-CBD cell populations was about 40% of that for cells with no bait in the CBD IBs (Fig. 5B). This result was consistent with the microscopic observations in Fig. 4A.

Finally, we attempted to purify cells with IB-localized red fluorescence using a single cell sorter, the FACSAria. For this experiment, equal amounts of cells with (pCN20-CBD) and without bait (pC20-CBD) were mixed, and the specific cells within a predetermined gate (dashed areas in Fig. 5A) were recovered. The collected cells were then analyzed by western blotting using an anti-GFP antibody (Fig. 5C). Lanes 1 and 2 show the control bands for bait-EGFP-CBD and EGFP-CBD, respectively. Before sorting, both proteins were observed in the cells (lane 3), whereas after sorting, the band corresponding to bait-EGFP-CBD was enriched in the recovered cells (lane 4), indicating selective sorting of cells with red fluorescent IBs due to protein-protein interactions between the bait and prey LZs.

### High physical stability of fluorescent IBs

**Figure 6:**
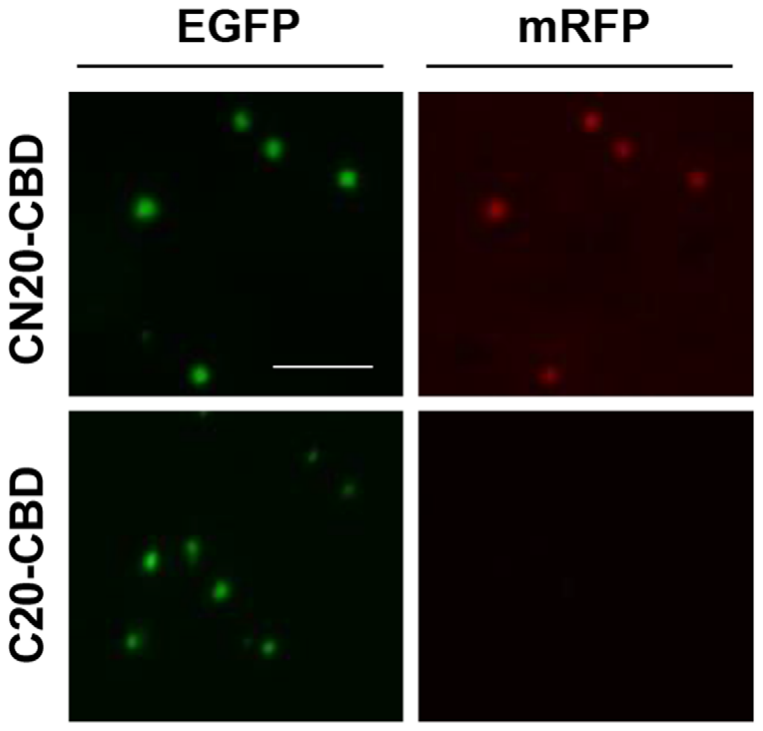
Comparison of fluorescent IBs purified from CN20-CBD and C20-CBD cells. Scale bar = 5 µm.

The functional IB particles were extracted from the CN20-CBD cells and the C20-CBD cells by sonication and washed twice with a solution containing 0.5% Triton X-100 detergent in a Tris buffer (50 mM Tris-HCl, pH 8.0 and 200 mM NaCl). Microscopic observation showed that the CN20-CBD IBs contained both green and red fluorescent IB particles, while the bait-less C20-CBD IBs contained only green fluorescent IBs because the prey-mRFP was washed out (Fig. 6). Therefore, the interactions in the active IBs were highly specific and were maintained during sonication and washing.

The physical stability of the active IBs was investigated using a zeta potential analyzer (Zetasizer Nano). The particle size was approximately 0.45–0.5 µm in diameter and the zeta potentials were estimated at approximately −56.8 mV. Zeta potentials larger than ±40 indicate that colloidal particles are stable in solution, while particles with a zeta potential smaller than ±30 tend to coagulate or flocculate easily [20]. Therefore, the IB particles in this study remained physically stable under both *in vivo* and *in vitro* conditions.

## Discussion

Synthetic biology, an emerging field, involves the design and construction of new genetic devices for use in research and industry [15], [21]. One successful device applied to metabolite production is the synthetic protein scaffold [16], [17]. When a heterologous or synthetic pathway is introduced, the host cell can suffer from flux imbalance, intermediate loss, and chemical toxicity [22]. Therefore, constructing synthetic scaffolds may improve the metabolite conversion rate by increasing the local enzyme concentration and reducing intermediate loss caused by diffusion or side reactions. In this regard, CBD IBs could be useful as a synthetic matrix in *E. coli* cells. The target proteins can be recruited to the synthetic IB matrix via bait and prey interactions between LZs (Fig. 1), which are a well-known domain consisting of only 30 amino acids. LZs such as E, K coil proteins have been used previously to immobilize active enzymes in polyhydroxybutyrate synthase IBs [14]. In this study, the affinities between LZs were controlled by changing the amino acid sequences. In addition, mRFP1 was used as a soluble target protein because it is easy to detect without cell disruption. Imaging and flow cytometric analyses showed that prey localization was dependent on the binding affinity between the bait and prey LZ proteins; the prey protein exhibited only marginal localization to the IBs when the K_D_ of the LZs was 1,000 µM (Fig. 3); as the K_D_ decreased, localization increased sharply and it reached a maximum level when the K_D_ was 8 or 20 µM. Eventually, we established a quantitative method to evaluate the localization of cytosolic proteins to IBs *in situ* by using LZs with different affinities (Fig. 3), which provides useful implications for the generation of synthetic matrices with designed compositions.

Localization of EGFP to IBs resulted in a large decrease in the fluorescence signal compared to the signal for soluble EGFP (Fig. S2), which is approximately half of the activity retention observed for catalytic enzymes in a previous study. The fluorescence intensity increased 2-fold when the particles were broken into smaller pieces by sonication (Fig. 2B). Based on literature reviews and our results in Fig. S4, the reason for the decreased fluorescence in the IBs is thought to be related with the scattering of the excitation light by the highly refractile surfaces of the IB particles [23], [24] and/or a shortened fluorescence lifetime in the densely packed environment [25]. In general, IBs are more dense (∼1.3 mg/mL) than any other cellular component.

Investigations of protein-protein interactions (PPIs) are crucial in modern biological science research [26], and there is growing interest in the development of high throughput technologies [18], [27]. In the method developed here, proteins with different affinity of LZs localized to IBs were quantitatively analyzed in living cells using flow cytometry (Fig. 5), while the E, K coil proteins in IB fractions was detected by electrophoretic methods after cell disruption in previous study [14]. Therefore, the current method can be applied usefully for high throughput screening of PPI inhibitors, comparisons of interacting protein partners, and engineering binding affinities in bacterial cells.

## Conclusions

Fluorescent proteins localized in IBs exhibited high intrinsic activity; however, their activity was somewhat suppressed when localized to IBs formed by fusion with the CBD from *C. fimi* exoglucanase. The signal intensity on microscopic images or in high throughput flow cytometry was dependent on the binding affinities of the interacting pairs. This controlled localization to IBs in living cells can be useful for the collective localization of cytosolic proteins in *E. coli* for sequential reactions. In addition, easy detection of protein localization to the IBs may provide a new platform for the rapid analyses of PPIs in bacterial cells.

## Supporting Information

Figure S1 **Construction of CN20-CBD (A) and C20-CBD (B).**
(TIF)

Figure S2 **Flow cytometric analyses of cells expressing EGFP and EGFP-CBD proteins.** The dark green and light green signals indicate cells expressing EGFP and EGFP-CBD, respectively.
(TIF)

Figure S3 **SDS-PAGE analysis of different leucine zipper proteins in** ***E. coli*** **cells (CN8-CBD, CN20-CBD, CN31-CBD, CN50-CBD, and CN1000-CBD).** The upper and lower arrows indicate the size of the NZ-EGFP-CBD and CZ-mRFP proteins, respectively.
(TIF)

Figure S4 **Side and forward scattering analyses of** ***E. coli*** **cells expressing EGFP (A) and EGFP-CBD (B).**
(TIF)

Table S1 **Primers used in this study.**
(TIF)
